# Determinants of maternal satisfaction with focused antenatal care services rendered at public health facilities in the West Shewa Zone, Central Ethiopia: A multicentre cross-sectional study

**DOI:** 10.3389/fgwh.2022.902876

**Published:** 2023-01-30

**Authors:** Gemechu Gelan Bekele, Benyam Seifu, Ephrem Yohannes Roga

**Affiliations:** Department of Midwifery, College of Medicine and Health Sciences, Ambo University, Ambo, Ethiopia

**Keywords:** pregnant women, satisfaction, ANC, West Shewa, Central Ethiopia

## Abstract

**Background:**

Every woman has the right to receive quality care during pregnancy. It is proven that antenatal care (ANC) reduces maternal and perinatal morbidity and mortality. The government of Ethiopia is also making intense efforts to increase the coverage of ANC. However, among pregnant women, the levels of satisfaction with the care provided are overlooked, as the percentage of women who complete all ANC visits is below 50. Therefore, this study aims to assess maternal satisfaction with ANC services rendered at public health facilities in the West Shewa Zone, Ethiopia.

**Methods:**

A facility-based cross-sectional study was conducted among women who were receiving ANC in public health facilities in Central Ethiopia between September 1 and October 15, 2021. A total of 411 women were selected using the systematic random sampling method. The questionnaire was pretested and the data were collected electronically using CSEntry. The collected data were exported to SPSS version 26. The characteristics of the study participants were described using frequency and percentage. Bivariate and multivariate logistic regression were used to identify the factors associated with maternal satisfaction with focused ANC service.

**Result:**

This study revealed that 46.7% [95% confidence interval (CI) (41.7%–51.6%)] of women were satisfied with ANC service. Factors such as the quality of the health institution [adjusted odd ratio (AOR) = 5.10, (95% CI: 3.33–7.75)], place of residence [AOR = 2.38, (95% CI: 1.21–4.70)], history of abortion [AOR = 0.19, (95% CI: 0.07–0.49)], and previous mode of delivery [AOR = 0.30, (95% CI: 0.15–0.60)] were significantly associated with women's satisfaction with focused ANC service.

**Conclusion:**

More than half of pregnant women who received ANC were dissatisfied with the service they received. This should be a cause for concern, as the level of satisfaction is lower than that of the findings of previous studies conducted in Ethiopia. Institutional variables, interactions with patients, and previous experiences of pregnant women have an impact on the level of satisfaction. Due attention should be paid to primary health and communication of health professionals with pregnant women to improve the levels of satisfaction with focused ANC service.

## Plain language summary

Despite a significant decline in maternal deaths globally, the daily loss of life among women continues to increase because of entirely preventable factors prior to, during, and following childbirth. Most maternal deaths primarily occur in low- and middle-income countries. Ethiopia is one of the countries with the highest maternal mortality rates in the world, estimated at 412 per 100,000 live births. This far outstrips the global goal of lowering maternal mortality to less than 70 per 100,000 live births by 2030. Prenatal care (ANC) provides a framework for taking significant healthcare initiatives such as disease prevention and health promotion.

The mother's satisfaction level with the antenatal care offered in the medical institution is clinically relevant and requires a painstaking assessment of the many aspects of healthcare. Although the level of satisfaction with maternal ANC services is crucial for the relevant stakeholders to advance mother and child health, little is known about these levels and their determinants in Ethiopia and the study area. Therefore, studying the satisfaction levels and their determinant factors will provide significant inputs for resource-poor settings in order to scale up maternal ANC service satisfaction. The finding of this result is important for effecting further improvements in maternal and child health.

## Introduction

Despite substantial advancement over the past few decades, ending preventable maternal deaths remains an unmet goal and one of the world's most critical concerns. Despite a 38% reduction in maternal deaths globally between 2000 and 2017, 810 women still lose their lives every day to mainly preventable causes before, during, and after childbirth. Low- and middle-income countries account for 94% of all maternal deaths (1, 2). Ethiopia has one of the highest maternal mortality rates in the world at 412 per 100,000 live births, well exceeding the global target of reducing maternal mortality to less than 70 per 100,000 live births by 2030 (3, 4).

One of the top priorities of Sustainable Development Goals (SDGs) is to lower maternal mortality (5). Antenatal care (ANC) remains one of the interventions that has the potential to significantly reduce maternal morbidity and mortality if properly provided (5–8). Focused ANC provides a framework for crucial medical procedures such as disease screening, disease prevention, and the management of pregnancy-related problems. In addition, it offers unique opportunities for the early detection and treatment of problems such as hypertension, gestational diabetes, anaemia, malaria, HIV, and other illnesses that might otherwise put the health of pregnant women and the developing fetus at risk (6, 7, 9, 10).

The women's level of satisfaction with the ANC service offered in the medical institution is clinically significant and requires an assessment of patients on several elements of healthcare, including organizational, interpersonal, and technical components (11–13). According to the literature, satisfaction with many aspects of prenatal care received enhances health outcomes, continuity of care, and treatment compliance (6–8, 14). To enhance the effectiveness and quality of prenatal care, the World Health Organization (WHO) suggests monitoring and evaluating maternal satisfaction with public health care services during pregnancy (7). The Ethiopian government has made great strides towards bringing health facilities closer to the community, mainly through constructing primary healthcare facilities and launching the health extension program, which could help in increasing the coverage of ANC services (15, 16). However, the experiences of pregnant women with the service provided are overlooked, as the percentage of women who complete receiving all ANC services is below 50. Moreover, many health facilities have started service provision without being properly equipped with the necessary equipment and qualified health personnel. For instance, of 80% of the facilities providing focused ANC, only 41% of them are able to provide high-quality ANC service (4).

Various studies have found varying levels of women's satisfaction with ANC services. These levels are as follows: approximately 69% of women in America (11), 90% in Kazakhstan (17), 98.5% in Nigeria (18), and 41.1% in Egypt (19). In Ethiopia, maternal satisfaction with ANC service varies by location, ranging from 30.3% to 83.9% (20, 21).

Studies have shown that factors such as the frequency of ANC visits, monthly income, educational level, age, pregnancy status, type of health facility, history of stillbirths, information on the potential risks of pregnancy, respectful maternity care, location, place of residence, provider of male sex services, occupation, and waiting time are important predictors of maternal satisfaction with ANC service (11, 19–25).

Coronavirus disease (COVID-19) has a major impact on the quality, satisfaction, and use of maternal and reproductive healthcare services, including ANC. Therefore, regular monitoring and evaluation of service performance is essential (26–28). The level of maternal satisfaction with ANC services is crucial for the relevant stakeholders to effect improvements in the area of mother and child health. However, most of the past studies conducted in Ethiopia assessed maternal satisfaction levels at single health facilities, and little is known about these levels and their determinants in present-day Ethiopia and the study area in particular. In light of this, this study aims to assess the level and determinants of maternal satisfaction with focused ANC services rendered at public health facilities in the West Shewa Zone in Central Ethiopia. The study's findings can aid policymakers, regulating authorities, health institutions, communities, and other groups working in the field of maternal, new-born, and child health to improve service quality.

## Materials and Methods

### Study design, area, and period

A facility-based cross-sectional study was conducted in public health facilities found in the West Shewa Zone, Central Ethiopia, from September 1 to October 15, 2021. The zone is located in the Oromia Regional State to the west of Addis Ababa, the capital city of Ethiopia. Ambo, the capital of the West Shewa Zone, is located 114 km away from Addis Ababa. According to information from the West Shewa Zonal Health Office in 2021, the total population in the zone is estimated to be 2,381,079, of which 1,214,350 are women. There are 9 government hospitals, 96 health centers, and 529 health posts in the zone. Women aged 15–49 are estimated to be 21.3%.

### Population

All mothers who have been receiving ANC service follow-up during the study period at public health facilities in the West Shewa Zone constitute the source population, while pregnant women who have been receiving ANC service in select public health facilities in the zone during this period form the study population. All pregnant women who resided in the study area for at least 6 months are included in the study.

### Sample size determination

The sample size was calculated using the single-population proportion formula, with the assumptions of a 95% (*α*/2 = 1.96) confidence level, 5% marginal error, 10% non-response rate, and a 53.8% level of antenatal care service satisfaction from Debra Tabor's previous study (22). Hence, after adding a 10% non-response rate, the final sample size was 420.

### Sampling procedure

Within the West Shewa Zone, where the study was done, there are 96 health centers and 9 public hospitals. A total of 3 hospitals and 20 health centers were randomly selected. In order to estimate the monthly calculable number of pregnant women for ANC service, 3-month ANC registration books at the chosen health institutions were reviewed. The total number of pregnant women who received ANC services in a given month was then divided by the calculated sample size of 420 to estimate the number of pregnant women from each public health facility. Finally, a systematic random sampling technique was used to select study participants.

There were 5,424 pregnant women who received focused ANC service in all public health facilities, which meant that, on average, 1,808 pregnant women received ANC service every month in all public health facilities. By proportionally allocating the calculated 420 sample size to the total number of pregnant women who received ANC services in each month (420/1,808), the sampling fraction became one-fourth. Then, every four pregnant women registered for ANC service throughout the data collection period were recruited from every health facility for this study.

### Data collection tool, procedure, and quality control

The data collection tool was adapted by reviewing similar kinds of literature to assess pregnant women's satisfaction with focused ANC services (10, 19–25) and others. The questionnaire was prepared in English and translated into the local language, Afan Oromo, and the translated version was used to collect the data. The questionnaire was designed in CSPRO version 7.3 software and exported to CSEntry for Android for electronic data collection. Data were collected by 13 BSC registered midwives, and five master’s holders supervised the data collection.

At Ginchi Primary Hospital, the questionnaire was pretested on 5% of the sample size, and any necessary corrections were made 1 week before the actual data collection time. A reproductive health expert assessed the content and face validity. The Cronbach alpha value, which was determined to assess the tool's internal consistency, was 0.86. Finally, data collectors and supervisors received 2 days of training on the study's objectives.

### Data processing and analysis

The CS-Entry android app was used to collect the data, which was then exported to SPSS version 25 for data analysis. Descriptive statistics were used for determining the frequency, percentage, mean, and standard deviation. The factors associated with the outcome variable were found using binary logistic regression. The multivariable logistic regression model included all explanatory variables that, in the bivariate analysis, had a significant relationship with the outcome variable. Crude and adjusted odds ratios with their 95% confidence interval (CI) were determined and a statistically significant association was found based on a *P*-value of less than 0.05. Multi-collinearity between the independent variables was also assessed using multiple linear regressions, and the variance inflation factor (VIF) for all variables was less than 10. The fitness of the logistic regression model was also evaluated using the Hosmer–Lemeshow statistic greater than 0.05.

### Operational definition

Women's satisfaction with focused ANC services meant that the women scored greater than or equal to the overall mean satisfaction score, from the total satisfaction with ANC service–related questionnaires (22).

## Results

### The demographic characteristics of the respondents

A total of 411 pregnant women receiving focused ANC services participated in this study, with the response rate being 97.8%. The mean age of the study participants was 25.8 years, with a standard deviation of ±4.6 years. A majority of the participants, 388 (94.4%), were of Oromo ethnicity. A total of 241 (60.1%) women were urban residents (Table 1).

**Table 1:** Socio-demographic characteristics of pregnant women receiving antenatal care at public health facilities in the West Shewa Zone, Central Ethiopia, 2021.

Variable	Category	Frequency (*N* = 420)	Percentage
Age in years	<20	53	12.9
	21–34	335	81.5
	>35	23	5.6
Marital status	Married	401	97.6
	Not married	10	2.4
Ethnicity	Oromo	388	94.4
	Amhara	17	4.1
	Gurage	6	1.5
Religion	Protestant	226	55
	Orthodox	160	38.9
	Others^a^	25	6.1
Mother occupation	Housewife	227	55.2
	Private employee	80	19.5
	Government employee	64	15.6
	Others^b^	40	9.7
Husband occupation	Not working	19	4.6
	Private employee	231	56.2
	Government employee	149	36.3
	Others^b^	12	2.9
Mothers’ education	No formal education	41	10
	Primary school	110	26.8
	High school	131	31.9
	College and above	129	31.4
Husband education	No formal education	34	8.3
	Primary school	59	14.4
	Secondary school	128	31.1
	College and above	190	46.2
Residence	Rural	241	60.1
	Urban	164	39.9
Average monthly income^c^	<1,380	67	16.3
	1,381–6,900	222	54
	>6,901	122	29.7

^a^ Wakefata and Catholic.

^b^ Students, daily labourers, and non-government organization employee.

^c^ WHO income classification for developing countries using Ethiopian Birr.

### Obstetric and reproductive health characteristics of the participants

The median age at pregnancy of the study participants was 22 years, with an interquartile range of 4 years. More than half (60.8%) of the participants, 250, were multigravida. A total of 203 (49.3%) pregnant women underwent ANC follow-up during their past pregnancy, while only 58 (23.2%) received postnatal care during their previous pregnancy. Forty-eight (11.7%) study participants had a history of abortion. Of the abortion cases, 42 (87.5%) were spontaneous in nature. A majority of the 244 participants (84.7%) delivered in a health facility. Nearly one-fifth (19.6%) of pregnant women, 49, had a history of medical disorders during their past pregnancy. More than half (60.1%) of them, 247, did not remember their last normal menstrual period during their pregnancy (Table 2).

**Table 2:** Obstetrics and reproductive health characteristics of pregnant women receiving ANC service at public health facilities in the West Shewa Zone, Central Ethiopia, 2021.

Variables	Categories	Frequency	Percentage
Gravida	Primigravida	161	39.2
	Multigravida	250	60.8
Number of children	No child	165	40.1
	1–3 children	219	53.3
	≥4 children	27	6.6
Place of last delivery	Home	44	15.3
	Health facility	244	84.7
Past illness during pregnancy	Pre-eclampsia	24	48.9
	HEG	17	34.7
	Gestation diabetes	8	16.3
	Heart disease	3	6.1
	Anaemia	5	10.2
	Others^a^	8	16.3
Number of past ANC visits	1–2	27	13.3
	≥3	176	86.7
Mode of delivery	SVD	178	61.8
	Vacuum/forceps	33	11.5
	Caesarean section	77	26.7
Pregnancy status	Intended	375	91.2
	Unintended	36	8.8

^a^ Epilepsy, goitre, tuberculosis.

### Women's satisfaction levels with focused ANC services

**Figure 1:**
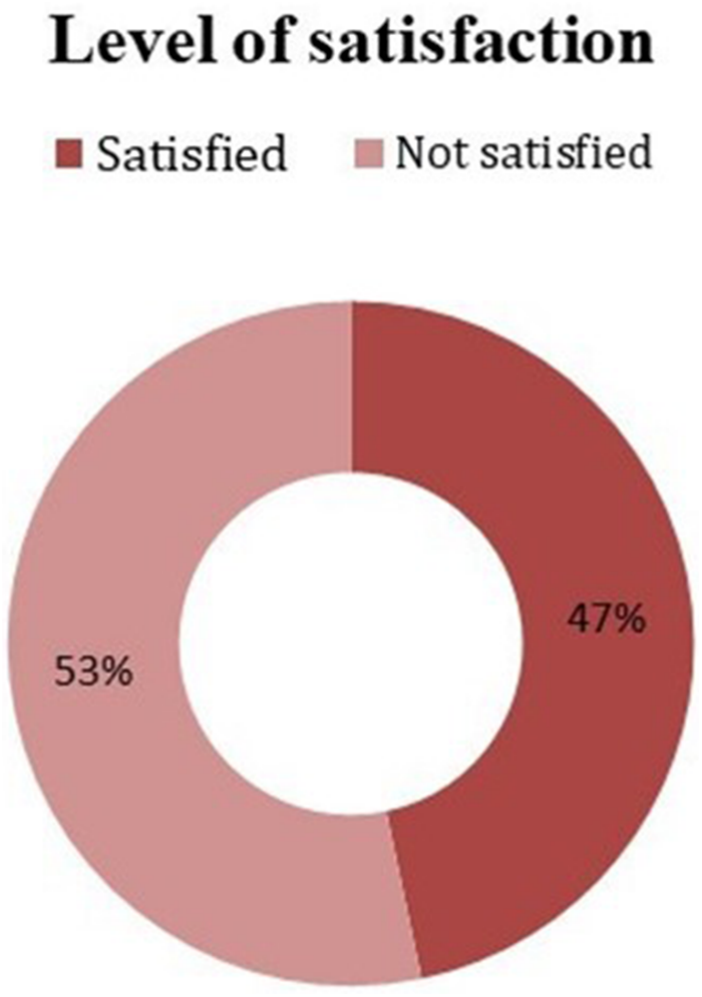
Satisfaction levels of pregnant women with focused ANC service at public health facilities of the West Shewa Zone, Central Ethiopia, 2021.

Pregnant women's levels of satisfaction were measured using 15 tools. Respondents were categorized as satisfied if they scored above the mean value and not satisfied if they scored below the mean value. Accordingly, 192 [46.7% (95% CI: 41.7–51.6)] pregnant women were satisfied with the focused ANC services offered at the public health facilities in the West Shewa Zone (Figure 1).

### Pregnant women's satisfaction with different domains of focused ANC services

The participants were highly satisfied with respectful maternal care (359, 87.3%) and supplementation of iron and folic acid (352, 85.6%).

However, 288 (70.1%) participants were dissatisfied with the way in which health professionals explained the rationale behind, and provided an interpretation of, laboratory investigation. About half (50.6%) of the women, 208, were dissatisfied with both auditory and visual privacy during the examination (Table 3).

**Table 3:** Level of client satisfaction with different domains of satisfaction in public health facilities of the West Shewa Zone, Central Ethiopia (*N = *411).

Components	Level of satisfaction		*P*-value
	Satisfied (%)	Not satisfied (%)	
Auditory and visual privacy during examination	203 (49.4)	208 (50.6)	0.001
Iron and folic acid supplementation	352 (85.6)	59 (14.4)	0.0001
Return home without receiving the service	171 (41.6)	240 (58.4)	0.409
Respectful maternity care	359 (87.3)	52 (12.7)	0.0001
Payment for the service received	360 (87.6)	51 (12.4)	0.0001
Willingness to give birth in this facility	307 (74.7)	104 (25.3)	0.0001
Received the needed care	286 (69.6)	125 (30.4)	0.001
Willingness to continue the follow-up (ANC) service	349 (84.9)	62 (15.1)	0.0001
Recommending others to be booked for ANC here	303 (73.7)	108 (26.3)	0.0001
Received counselling on the potential risks of pregnancy	197 (47.9)	214 (52.1)	0.0001
Waiting time to receive the care	282 (68.6)	129 (31.4)	0.0001
Distance from nearby facility	317 (77.1)	94 (22.9)	0.001
Rationale and interpretation of investigation	123 (29.9)	288 (70.1)	0.0001
History taking during ANC visit	262 (63.7)	149 (36.3)	0.249
Health education and information	246 (59.9)	165 (40.1)	0.0001
Overall satisfaction	46.70%	53.30%	

### Determinants of maternal satisfaction with focused ANC services

Both bivariate and multivariable binary logistic regression analyses were conducted to identify the determinants of maternal satisfaction with focused ANC services.

Multivariable logistic regression analysis showed that the odds of satisfaction were 5 times higher among pregnant women who availed of ANC service at a hospital compared with those utilizing the service at a health center [AOR = 5.10, (95% CI: 3.33–7.75)]. Urban residents were 2.38 times more likely to be satisfied with ANC service [AOR = 2.38, (95% CI: 1.21–4.70)]. The history of abortion was also a significant predictor of maternal satisfaction. Women with a history of abortion were 19% less likely to be satisfied with ANC service [AOR = 0.19, (95% CI: 0.07–0.49)]. Moreover, the previous mode of delivery was also found to be associated with satisfaction with ANC service. Women whose previous mode of delivery was the caesarean section had lower odds of satisfaction compared with those with spontaneous vaginal delivery [AOR = 0.25, (95% CI: 0.12–0.54)] (Table 4).

**Table 4:** Determinants of maternal satisfaction with ANC service among pregnant women receiving ANC at public health facilities in the West Shewa Zone, Central Ethiopia, 2021.

Variables	Overall satisfaction with ANC		COR (95% CI)	AOR (95% CI)	*P*-value
	Satisfied (%)	Dissatisfied (%)			
Age of participants					
<20	22 (41.5)	31 (58.5)	2.56 (0.82–7.92)	3.49 (0.67–18.2)	0.138
21–34	165 (49.3)	170 (50.7)	3.49 (1.27–9.63)	3.12 (0.85–11.5)	0.086
≥35	5 (21.7)	18 (78.3)	1	1	
Level of health institution					
Hospital	137 (65.6)	72 (34.4)	5.27 (3.01–9.40)	5.10 (3.33–7.75)	0.0001
Health centre	55 (27.2)	147 (72.8)	1	1	
Place of residence					
Rural	99 (40.1)	148 (59.9)	1	1	
Urban	93 (56.7)	71 (43.3)	1.96 (1.31–2.92)	2.38 (1.21–4.70)	0.013
Average monthly income (in Ethiopian Birr)					
<1,380	27 (40.3)	40 (59.7)	1	1	
1,381–6,900	90 (40.5)	132 (59.5)	1.01 (0.58–1.76)	0.99 (0.43–2.33)	0.992
>6901	75 (61.5)	47 (38.5)	2.36 (1.29–4.35)	2.69 (0.97–7.42)	0.057
Mode of delivery of the last pregnancy					
SVD	100 (56.2)	78 (43.8)	1	1	
Vacuum/forceps	14 (42.4)	19 (57.6)	0.58 (0.27–1.22)	0.64 (0.26–1.57)	0.327
Caesarean section	21 (27.3)	56 (72.7)	0.29 (0.16–0.52)	0.30 (0.15–0.60)	0.001
Antenatal care follow-up during last pregnancy					
No	29 (34.1)	56 (65.9)	1	1	
Yes	106 (52.2)	97 (47.8)	2.11 (1.25–3.57)	1.86 (0.97–3.54)	0.060
History of abortion					
No	183 (52.1)	168 (47.9)	1	1	
Yes	9 (15)	51 (85)	0.16 (0.08–0.34)	0.19 (0.07–0.49)	0.001
Place of last delivery					
Home	11 (25)	33 (75)	1	1	
Health facility	124 (50.8)	120 (49.2)	3.1 (1.49–6.41)	2.39 (0.99–5.71)	0.050

## Discussion

This study assessed pregnant women's satisfaction with focused ANC services and the associated factors in public health facilities in the West Shewa Zone, central Ethiopia. Accordingly, women's satisfaction with focused ANC services was found to be 46.7% [95% CI: (41.7–51.6)], which was lower than that of most previous studies conducted in different places of Ethiopia, for example, 60.4% in Jimma Town (29), 79.2% in Hawassa city (30), 74% in Hossana (25), 70.3% in the Harari region (23), and 68% in Southwest Ethiopia (31). It was also lower than that of the studies done in Nigeria and Kazakhstan, where 90% of pregnant women were satisfied with ANC services (17, 18).

Nonetheless, the satisfaction level in this study was higher than that in the ones conducted in the Bursa district (33%) and Northwest Ethiopia, 30.3% (20, 32). This discrepancy could be due to differences in the study period, socio-demographic characteristics, and the measurement scale used to determine the satisfaction levels. Moreover, the variation might be due to the difference in the study settings, as some studies included only health centers (21, 30, 32), while this study included both hospitals and health centers.

The odds of maternal satisfaction with ANC service were 5 times higher among pregnant women utilizing the service at hospitals compared with those receiving the service at health centers. Likewise, the study conducted in the Harari region, eastern Ethiopia (23), revealed that the levels of satisfaction were higher among those who received the service within the hospital than those who availed it in health centers. This could be due to the fact that hospitals have more resources, infrastructure, human power, medical equipment, and other facilities compared with health centers (33).

Place of residence was also found to be a significant determinant factor associated with maternal satisfaction with ANC service. Urban residents had 2.38 times higher odds of satisfaction with the service than rural residents. This was supported by previous studies conducted in the Tullo and Chencha districts of Ethiopia (34, 35). This was also supported by the finding of the Ethiopian demographic health survey, which revealed that urban women were more likely (85%) than rural women (70%) to receive ANC service from a skilled provider (3). This could be attributed to the fact that rural dwellers travel a long distance to reach health facilities and are forced to wait for a long time to receive the desired care (36). There could also be discrepancies between urban women and rural residents in terms of communication (37).

Compared with their counterparts who had normal deliveries, pregnant women who previously had an abortion had a 19% lower likelihood of being satisfied with ANC service. The potential explanation for this is that they anticipate receiving high-quality care for their current pregnancy in order to avoid issues that occurred during their previous pregnancy. There is a high likelihood that they would be unhappy with ANC service if they feel that it falls short of their expectations. Finally, this study revealed that women whose previous mode of delivery was caesarean section had 30% lower odds of satisfaction compared with those with spontaneous vaginal delivery. Caesarean birth entails the physiologic stresses of anaesthesia, a major surgical procedure, physical recovery, and postoperative complications. Caesarean birth adds additional stress, including the stress of surgery. Women who experience caesarean delivery have reported feelings of depression, anxiety, guilt, less satisfaction with the birth experience, loss of control, and loss of self-esteem (38). Furthermore, they may expect high-quality care for the early detection of problems in order to avoid harming their fetus and having to undergo a repeat caesarean section.

### Strengths and limitations of the study

Social desirability bias might have affected the quality of the collected data. However, interviews were conducted after women received care upon exit by non-staff members to minimize bias. The other possible limitation could be that pregnant women were included irrespective of past obstetric history and gestational age, because of which primigravida mothers and those on their first visit might have faced difficulty judging some domains of satisfaction at first exposure. Moreover, the quantitative nature of the study could be another limitation. Therefore, we recommend that readers consider this limitation while citing and interpreting the findings of this study.

## Conclusion

Only approximately 47% percent of the participants felt satisfied with the focused ANC services rendered at public health facilities in Central Ethiopia. The participants were highly satisfied with respectful maternal care and iron and folic acid supplementation. However, they were highly dissatisfied with the way health professionals explained the rationale behind and provided an interpretation of laboratory investigation and privacy during examination. Moreover, factors such as the type of health institution, place of residence, history of abortion, and mode of delivery during last pregnancy were found to be significantly associated with maternal satisfaction with ANC service. Therefore, health facilities and the stakeholders concerned need to take necessary measures to scale up the satisfaction levels of pregnant women as these are also a reflection of standard care. They also ought to focus on infrastructure within the facilities as well as manpower to minimize waiting time and provide high-quality service, including counseling on the potential risks of pregnancy.

## Data Availability

The original contributions presented in the study are included in the article/Supplementary Material, and further inquiries can be directed to the corresponding author.
